# Role of Microbiota and Tryptophan Metabolites in the Remote Effect of Intestinal Inflammation on Brain and Depression

**DOI:** 10.3390/ph11030063

**Published:** 2018-06-25

**Authors:** Barbora Waclawiková, Sahar El Aidy

**Affiliations:** Department of Molecular Immunology and Microbiology, Groningen Biomolecular Sciences and Biotechnology Institute (GBB), University of Groningen, Nijenborgh 7, 9747 AG Groningen, The Netherlands; b.waclawikova@rug.nl

**Keywords:** microbiota, kynurenine pathway, serotonin, inflammation, gut motility

## Abstract

The human gastrointestinal tract is inhabited by trillions of commensal bacteria collectively known as the gut microbiota. Our recognition of the significance of the complex interaction between the microbiota, and its host has grown dramatically over the past years. A balanced microbial community is a key regulator of the immune response, and metabolism of dietary components, which in turn, modulates several brain processes impacting mood and behavior. Consequently, it is likely that disruptions within the composition of the microbiota would remotely affect the mental state of the host. Here, we discuss how intestinal bacteria and their metabolites can orchestrate gut-associated neuroimmune mechanisms that influence mood and behavior leading to depression. In particular, we focus on microbiota-triggered gut inflammation and its implications in shifting the tryptophan metabolism towards kynurenine biosynthesis while disrupting the serotonergic signaling. We further investigate the gaps to be bridged in this exciting field of research in order to clarify our understanding of the multifaceted crosstalk in the microbiota–gut–brain interphase, bringing about novel, microbiota-targeted therapeutics for mental illnesses.

## 1. Introduction

The complex communities of the microbiota that inhabit the mammalian gut have a significant impact on the health of their host. These gut bacteria has coevolved with the human body to perform numerous beneficial functions ranging from being simple fermenters of food to having profound effects on the host immune development, metabolism and food preferences, brain development, stress responses, pain, and behavior [1,2,3,4,5]. Consequently, disruptions or alterations in this resilient relationship are a significant factor in many diseases such as inflammatory gastrointestinal diseases and neuropsychiatric disorders, including depression [1,6,7,8]. 

Depression is a severe neuropsychiatric disease with multiple comorbidities in play. According to the World Health Organization (WHO), this longstanding mental disorder affects more than 300 million people of all ages worldwide [9]. Moreover, it is the leading cause of disability in modern society, and approximately one million people suffering from depression commit suicide every year [9]. It is widely recognized now that depression is closely linked with inflammation, and disrupted serotonergic systems throughout the human body, including the gut [10,11,12,13,14,15,16]. In fact, in a state of inflammation, not only high levels of proinflammatory cytokines are produced but also altered levels of neurotransmitters, such as serotonin, a derivative of tryptophan metabolism, are detected in the gut [17,18,19]. The presence of vast majority of bodily serotonin and immune cells in the gut in close proximity to the trillion of the gut-associated microbes implies the gut microbiota is likely to be an orchestrator in this multifaceted crosstalk between inflammation, serotonin, and depression, as will be discussed in this review article. 

## 2. Gastrointestinal Inflammation and Depression

**Figure 1:**
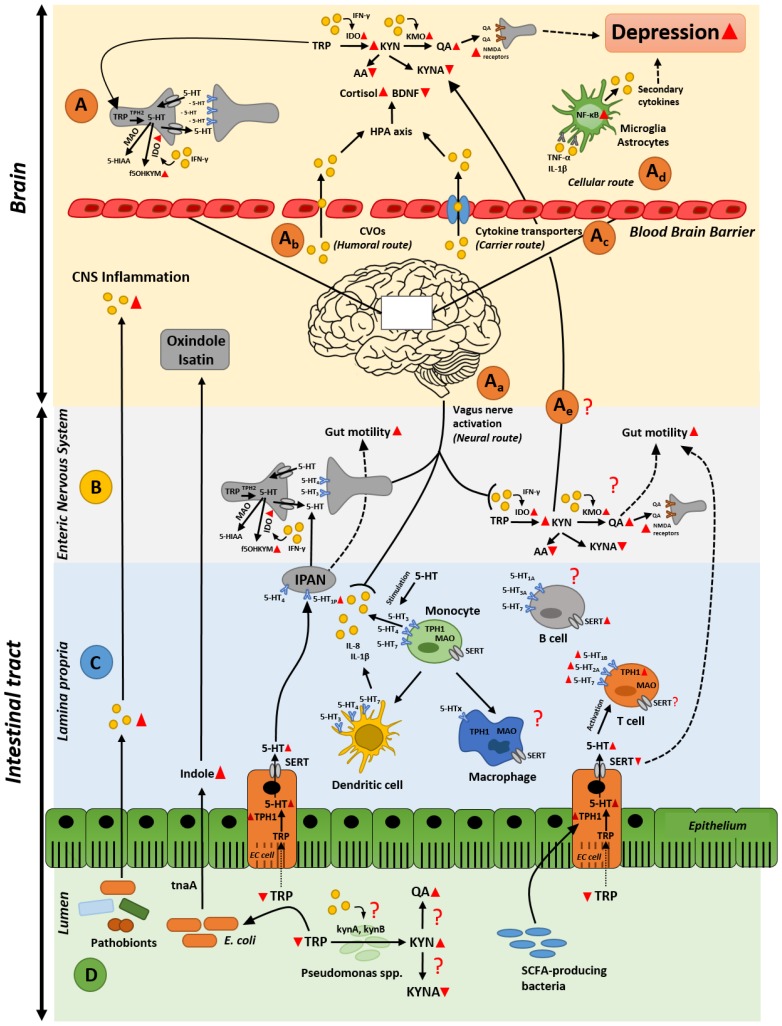
Gut microbiota remotely influences brain and depression. Potential routes by which the gut microbiota could govern the comorbidity of gut inflammation, disruption in tryptophan metabolism, and induction of depression. (**A**) Signaling mechanisms in the brain stimulated by inflammatory molecules in the gut; (**B**) molecular mechanisms by which the enteric nervous system affects gut motility and tryptophan metabolism during intestinal inflammation; (**C**) possible alterations of serotonin signaling in lamina propria resulting in gut hypermobility and inflammatory response; (**D**) influence of the gut microbiota and its secreted compounds on disruption on tryptophan metabolism and gut inflammation. Red triangles represent decreased/increased production or expression. Red question marks indicate missing links in this multi-faceted crosstalk. Dotted lines depict effects on gut motility. Abbreviations: 5-HIAA = 5-hydroxyindoleacetic acid; 5-HT = serotonin; 5-HT_x_ = serotonin receptors; AA = anthranilic acid; BDNF = brain-derived neurotrophic factor; CVOs = circumventricular organs; EC cell = enterochromaffin cell; HPA axis = hypothalamus-pituitary adrenal axis; IDO = indoleamine-2,3-dioxygenase; IFN-γ = interferon γ; IPAN = intrinsic primary afferent neuron; KMO = kynurenine-3-monooxygenase; KYN = kynurenine; KYNA = kynurenic acid; MAO = monoamine oxidase; NMDA receptors = *N*-methyl-d-aspartate receptors; QA = quinolinic acid; SCFAs = short-chain fatty acids; SERT = serotonin transporter; TNF-α = tumor necrosis factor α; TPH1 = tryptophan hydroxylase; TRP = tryptophan; f5OHKYM = formyl-5-hydroxykynuramine; kynA = tryptophan-2,3-dioxygenase from Pseudomonas spp.; kynB = kynurenine formamidase from Pseudomonas spp.; tnaA = tryptophanase.

In a state of intestinal inflammation, the immune system responds by producing various proinflammatory cytokines and metabolites, several of which are detected in the systemic blood circulation [20]. The fact that these molecules can cross the blood–brain barrier suggests that they can signal to the brain to ultimately result in serious changes in behavior [21]. Several routes, by which cytokines and metabolites present in the gut can influence the brain and behavior, have been described; (1) neural, (2) humoral, (3) cellular, and (4) carrier route [22] (Figure 1A). In the neural route, afferent nerves, such as the vagus nerve, are involved. Vagal nerves are activated by proinflammatory cytokines and other metabolites released by immune cells, neurons or intestinal bacteria during intestinal inflammation or infections [23] (Figure 1A_a_). This cascade leads to activation of the hypothalamus–pituitary–adrenal axis, thus increased levels of cortisol (stress hormone) and decreased levels of brain-derived neurotrophic factor [21]. The humoral route involves signaling via the circumventricular organs (CVOs), which have been described as a way by which leukocytes can reach the central nervous system [24]. CVOs are areas in the brain that lack an intact blood–brain barrier, thus allow molecules with limited access to the brain to migrate [25] (Figure 1A_b_). The carrier route includes cytokine transporters at the blood–brain barrier, where circulating cytokines can access the brain via the energy- and carrier-dependent active transport system or via no energy-dependent carrier-mediated facilitated diffusion system, commonly called saturable transport systems [26] (Figure 1A_c_). Finally, the cellular route involves cytokine receptors, such as receptors for tumor necrosis factor α (TNF-α) and interleukin (IL)-1β, expressed on non-neuronal cells in the brain, such as microglia and astrocytes [27,28,29]. Binding of TNF-α and IL-1β to their receptors in the brain activates cerebral NF-κB signaling pathway and induces the production of secondary cytokines, which can promote a depressed mood [29,30] (Figure 1A_d_). Indeed, proinflammatory cytokines, such as interferon-γ (IFN-γ), IL-2, TNF-α, and inflammatory markers such as C reactive protein (CRP), have been linked to higher risk of depression [31,32]. All together, these pathways show a number of sophisticated signaling mechanisms in the brain, which when stimulated by molecules produced during intestinal inflammation could lead to altered brain functionality, potentially leading to progression of depression. 

### 2.1. A Gut Perspective on the Role of Tryptophan Metabolites in Depression 

Tryptophan is an essential amino acid, derived from the diet [33]. Apart from its role in protein synthesis, tryptophan and its metabolites are associated with numerous physiological functions, such as immune homeostasis, but also with inflammatory response [34]. Once absorbed in the gut, tryptophan can cross the blood–brain barrier to participate in serotonin synthesis [35]. However, there are many other pathways through which tryptophan can be readily metabolized in the gut, thereby influencing its availability to pass the blood–brain barrier. Among these pathways are the kynurenine [36] and the serotonin synthesis pathways within the gut [37,38]. Kynurenine and serotonin are vital signaling molecules in immune response and gut–brain communication [34,39,40]. Most of the digested tryptophan (about 90%) is metabolized along the kynurenine biosynthesis pathways [41], while only approximately 3% is metabolized into serotonin throughout the body, and the rest is degraded by the gut microbiota to produce indole and its derivatives [42]. This implies a strong competition between serotonin and the first downstream metabolites from the kynurenine pathway, kynurenine, for the available tryptophan, as described below. In inflammatory conditions, more kynurenine is produced on the expenses of serotonin [43,44], which, if happens also in the brain, results in behavioral changes including persistent sadness, loss of interest, and decreased energy levels [45] (Figure 1A). 

### 2.2. Gut Inflammation-Induced Kynurenine Biosynthesis; Possible Cause of Altered Kynurenine Pathway in the Brain during Depression?

Tryptophan forms kynurenine via the rate-limiting enzyme, indoleamine-2,3-dioxygenase (IDO) enzyme, found ubiquitously in all tissues, including the gut, and tryptophan-2,3-dioxygenase (TDO), which is localized in the liver [41]. The activity of TDO and IDO is uniquely induced by different stimuli; while TDO is induced by stress-elevated glucocorticoids, such as cortisol [46], IDO is induced during intestinal inflammation [47] by proinflammatory stimuli, with interferon γ (IFN-γ) being the most potent inducer [48]. Induction of IDO results in a shift in the tryptophan metabolism towards the production of kynurenine and its downstream metabolites; kynurenic acid, anthranilic acid, and quinolinic acid rather than serotonin synthesis [47,49] (Figure 1A,B). Moreover, activated IDO accelerates the degradation of serotonin into formyl-5-hydroxykynuramine [50,51]. Degradation of serotonin yields reactive oxygen species byproducts and subsequently inflammation [52]. This further intensifies serotonin deficiency, leading to the disruption in neurotransmission and consequently causes depression. 

In contrast to kynurenic acid and quinolinic acid, kynurenine and anthranilic acid can cross the blood–brain barrier via the saturable transfer [53]. This suggests that inflammatory-induced altered levels of kynurenine in the gut may transfer to the blood circulation and ultimately to the brain, resulting in altered levels of kynurenine and its metabolites, kynurenic acid, and quinolinic acid (Figure 1A). In fact, during inflammation, the enzymes of the kynurenine pathway are activated leading to changed production of kynurenic and quinolinic acids. Depression is believed to arise from the excessive production of the neurotoxic quinolinic acid together with a reduction in kynurenic acid [54]. Reduced levels of kynurenic acid have been correlated with severe depressive and suicidal symptoms [55,56], and decreased blood levels of this molecule has been detected in the patients with major depressive disorder [57]. Quinolinic acid is a neurotoxic agent, and its production is significantly enhanced by proinflammatory cytokines through their stimulation of the rate limiting step enzyme in the quinolinic acid pathway, kynurenine-3-monooxygenase (KMO) enzyme [58,59]. For instance, levels of quinolinic acid in the cerebrospinal fluids of suicide attempters showed around 300% increase compared to healthy controls [60]. In the brain, quinolinic acid acts as an agonist of *N*-methyl-d-aspartate (NMDA) receptors, which play a key role in the regulation of synaptic function [61]. Activation of NMDA receptors upon binding to quinolinic acid has been described to be another mechanism involved in promotion of depression [49]. Specifically, when microglia are stimulated by proinflammatory cytokines, glutamate, another agonist of NMDA receptors, and the main excitatory neurotransmitter in the central nervous system, is released leading to additional activation of NMDA receptors [61]. Therefore, quinolinic acid alone or in combination with glutamate, can enhance NMDA receptor activation and subsequently lead to depression [49]. Intriguingly, a high proportion of the enteric neurons in the gut express the NMDA-type glutamate receptors [62,63]. In line with these observations, previous data have suggested that enhanced activation of NMDA receptors maybe involved in altered inflammation-linked motility and in inflammatory-induced nociception [64,65] as a remote consequence of intestinal inflammation on the brain [66] (Figure 1B). In contrast to quinolinic acid, kynurenic acid is an indigenous antagonist of the enteric NMDA receptors, thereby suppressing the hypermobility of the gut associated with the activated NMDA receptors and excitability of the enteric neurons during an intestinal inflammatory response. Collectively, the current data support an effect of intestinal inflammation on redirecting the tryptophan metabolic pathway towards kynurenine rather that serotonin biosynthesis. Kynurenine metabolites have a profound effect on the enteric nervous system and intestinal motility alteration. Whether altered gut motility can stimulate a state of depression and whether changes in intestinal kynurenine metabolism could be the source of altered levels of these metabolites in the brain, warrant more investigation. 

### 2.3. Intestinal Inflammation and Disrupted Serotonin Signaling System: From Alterated Gut Functionality to Development of Depression

Serotonin is a key-signaling regulator that modulates a wide range of effects on host physiology, including the control of gut motility, secretory reflexes, platelet aggregation, regulation of immune responses, and regulation of mood and behavior [67]. Once tryptophan is absorbed in the gut, it crosses the blood–brain barrier to be partially metabolized into serotonin in the raphe nuclei within the brain stem [34]. However, the majority (~95%) of serotonin in the body is synthesized, stored, and released in the gut, mainly from a subset of enteroendocrine cells called enterochromaffin cells in the intestinal mucosa [19]. The small amount of serotonin that is not in enterochromaffin cells is in the enteric nervous system, in particular, the myenteric plexus, which contains descending serotonergic interneurons [68] (Figure 1B). 

Enterochromaffin cells, also known as epithelial sensory transducers, secrete serotonin in response to mucosal stimuli, such as microbiota metabolites as discussed below. Once synthesized, serotonin is secreted in the lamina propria, where it has access to the nerve fibers. This implies a large amount of serotonin is secreted in the extracellular space. Thus, to avoid receptors’ desensitization by their contact with excessive amounts of serotonin, which is toxic [69], serotonin overflow must be efficiently controlled. One important player in serotonin uptake by gut epithelial cells, thus serotonergic termination, is the Na^+^/Cl^−^ dependent, serotonin transporter (SERT). SERT, is a recently crystallized protein [70] comprised of 12 transmembrane domains, and is a member of a large superfamily of sodium/chloride dependent transporters, which also contain transporters for other neurotransmitters, such as dopamine and norepinephrine [71]. 

Secreted serotonin mediates its actions via several receptor subtypes [72], where it has been observed to affect epithelial cells’ proliferation and secretion [73], but mainly acts as a regulator of the gut motility. Secretion of serotonin by enterochromaffin cells activates intrinsic primary afferent neurons (IPANs) in the submucosal plexus via its action on 5-HT_1P_ receptor. These cells initiate peristaltic and secretory reflexes, which influences gut motility (Figure 1C). Moreover, intestinal serotonin activates extrinsic sensory nerves via its action on 5-HT_3_, which are postsynaptic receptors found on the terminals of extrinsic sensory neurons terminal in the gut and transmit noxious signals to the brain [74] (Figure 1B). Though it does not initiate peristaltic movement, 5-HT_3_ conveys any kind of change in gut motility to the brain via its presence on myenteric IPANs and in the myenteric plexus, where they mediate fast excitatory neurotransmission [75]. Similarly, 5-HT_4_ receptors themselves do not initiate peristaltic reflexes, but because of their location at the terminals of submucosal IPANs, at synapses within the myenteric plexus, and at the neuromuscular junction, stimulation of 5-HT_4_ receptors is critical for these reflexes [76]. 5-HT_4_ receptors work through stimulating the production of the neurotransmitters acetylcholine and calcitonin gene-related peptide, which enhances the spread of stimuli around and through the gut wall, to ultimately enhance and maintain a normal gut motility [77,78]. 

The strong link between inflammation, and disruptions of serotonin metabolism has been well established. Immune cells including lymphocytes, mast cells, dendritic cells and monocytes have all been reported to express SERT, serotonin receptors and enzymes involved in the production and metabolism of serotonin [79,80] (Figure 1C). T lymphocytes express the main components of serotonin metabolism, i.e., tryptophan hydroxylase (TPH), the first rate limiting enzyme involved in serotonin production, SERT, monoamine oxidase (MAO) [80], which breaks down serotonin into its metabolite 5-HIAA, and 5-HT receptors [80]. While resting, naïve T cells express very little TPH1, the TPH isoform present in the intestinal enterochromaffin cells, where intestinal serotonin is synthesized, activated T cells show approximately 30-fold higher expression of TPH1, suggesting increased levels of serotonin in activated T cells [81], and 5-HT receptors, including 5-HT_1B_, 5-HT_2A_, and 5-HT_7_ receptors [81]. However, expression of SERT in T cells is still questionable; León-Ponte et al. shows that neither naïve nor activated T cells express high-affinity SERT [81], however another study claims that SERT is present in T cells membranes [82] (Figure 1C). Thus, these contradictory conclusions warrant further investigation. B lymphocytes are also known to express 5-HT receptors, including 5-HT_1A_, 5-HT_2A_, 5-HT_3A_ and 5-HT_7_ [80], and activated B cells exhibit a significant increase in SERT expression [83] (Figure 1C). Whether B cells express other components of serotonin machinery and thus influencing serotonin signaling, it is still unknown. Like T cells, monocytes, the immature leukocytes that eventually differentiate into macrophages or dendritic cells, express the complete set of components needed for serotonin production [80]. Dendritic cells, have also been found to mediate the release of proinflammatory cytokines, IL-1β and IL-8 via 5-HT_3_, 5-HT_4_, and 5-HT_7_ receptor subtypes [84]. In fact, serotonin has been demonstrated as an important regulator of the immune system. For example, serotonin has been described to modulate proinflammatory cytokines production in human monocytes via stimulation of different 5-HT receptor subtypes, particularly 5-HT_3_, 5-HT_4_, and 5-HT_7_ receptors [85] (Figure 1C). Interestingly, deletion of 5-HT_4_ receptors in mice results in inflammatory response, slowed colonic motility, and behavioral abnormalities [86,87]. Similarly, reduced expression of SERT and subsequent altered serotonin levels, have been associated with different inflammatory and diarrheal disorders [15,16,88]. Targeted deletion of the SERT in mice led to increased colonic motility and increased water in stools [89] in a similar manner to that observed in inflammatory bowel disorders, where SERT expression is also reduced [18,90]. That the altered structure or expression of SERT leads to disrupted serotonin transmission [91], the current data point to a strong link between intestinal inflammation, disruption of serotonin signaling and the consequent alteration in gut motility, and development of depression. Whether the altered gut motility [14,16,88,89,92,93] is the driving factor in inducting depression in this cascade is unclear. One plausible mechanism is via the gut motility-mediated changes in the microbial population complexity, which might exert detrimental effects on enteric and central neurons leading to a state of depression.

## 3. Microbiota as an Orchestrator in the Crosstalk between Inflammation and Serotonin Imbalances

The presence of vast number of gut microbiota in close proximity to serotonin and immune cells in the gut, makes it plausible to consider these bacteria as a conductor in the orchestra of intestinal inflammation and serotonin, to remotely result in a state of altered mood and depression in the brain (Figure 1).

### 3.1. Gut Microbiota and Intestinal Immune (Hyper)-Stimulation

It is well-established that the gut microbiota plays a critical role in both innate and adaptive immunity, where it mediates the formation, maturation, and function of several immune cells [94]. Interactions between the gut bacteria and gut mucosa regulate the production of numerous proinflammatory cytokines [95,96,97]. Several species within the gut bacteria have been shown to be essential in the development and maturation of the immune response. For example, a monocolonization of germ-free mice with the ubiquitous gut bacterium, *Bacteroides fragilis*, shows immunomodulatory activities of this bacterium, including correction of T cell deficiencies and T_H_1/T_H_2 imbalances [98]. Segmented filamentous bacteria, the epithelial-associated bacteria, stimulate the maturation of proinflammatory IL-17A-producing T helper 17 (T_H_17) cells in the mouse small intestine [99,100]. Though considered as commensals, these bacteria have pathogenic properties and are referred to as pathobionts due to their capacity to induce a profound inflammatory state if an imbalance within the microbial population (also known as dysbiosis) occurs [101]. Microbial dysbiosis has been strongly linked to inflammatory bowel disease, a comorbidity of anxiety and depression [102]. Increased relative abundance of *Escherichia coli* and *Enterococcus faecalis* have been described to induce intestinal inflammation and bacterial-antigen specific cytokine production (IFN-γ and IL-4) in a well-characterized murine colitis model *IL10^−/−^* [103]. Several *Bacteroides* genera have been recognized to be important for induction of inflammatory bowel disease in *IL-10r2^−/−^* × *Tgfbr2^−/−^* mouse colitis model [104]. *Klebsiella pneumoniae* and *Proteus mirabilis*, has been positively correlated with colitis in *Tbx21^−/−^* × *Rag2^−/−^* mouse inflammatory bowel disease model [105]. A common resident of the human mouth and gut, *Fusobacterium nucleatum*, when isolated from the inflamed gut of Crohn’s disease patients evoked significantly greater TNF-α gene expression [106]. Finally, in experimental autoimmune encephalomyelitis, a mouse model of multiple sclerosis, germ-free or antibiotic-treated mice exhibited reduced inflammation and disease scores compared to conventional mice, suggesting a role for gut microbes on peripheral immune response, leading to brain inflammation [107,108]. Overall, these data suggest gut microbiota as an important immunomodulatory player in the gut–inflammation–brain crosstalk (Figure 1D).

### 3.2. Gut Microbiota and Serotonin Production 

Recently, it has been shown that gut microbiota plays an important role in the regulation of the host serotonin levels [109]. Particular microbial metabolites, namely short chain fatty acids, have been shown to promote serotonin production from enterochromaffin cells in the epithelia via induction of TPH1 gene expression [109,110] (Figure 1D), most likely, due to their acidic pH. The effect of gut microbiota on intestinal serotonin levels expands beyond the gut. Plasma serotonin levels were in germ-free mice compared to conventional mice [111]. On the other hand, levels of hippocampal serotonin were significantly increased in germ-free and colonized germ-free mice compared to conventional mice [112]. However, a causation of differences in serotonin levels in germ-free mice still needs to be explained. Yano et al., further showed that SERT expression is increased in germ-free mice, suggesting its regulation by gut microbiota [109]. Indeed, SERT genotype has been linked to altered gut microbiota composition in young rats [88], where SERT knock out rats showed imbalanced microbial community dominated by members of the gut microbiota previously reported to be associated with a state of intestinal inflammation, and brain disorders including multiple depressive disorders [106,113,114,115,116,117,118]. Of note, the observed microbial imbalance was magnified when young rats were exposed to another stimulus, maternal separation [88], implying that the absence or domination of certain bacterial members in the gut of early-life stressed individuals may represent risk factors for the development of depression during later life stages. 

Gut produced serotonin has been the target of several antidepressants, such as fluoxetine, which block its transport into the plasma via targeting SERT, thus named selective serotonin reuptake inhibitors (SSRIs). Administration of these antidepressants has been also successfully used as a treatment for gastrointestinal diseases, such as motility disorder and gastrointestinal bleeding [92,119], confirming comorbidity of these disorders, but exert a puzzling effect on the intestinal bacterial composition. Fluoxetine has an antimicrobial activity against Gram-positive bacteria such as *Staphylococcus* and *Enterococcus* and some anaerobic bacteria such as *Clostridium difficile* and *Clostridium perfringens* [3,120,121,122]. Similarly, Gram-negative bacteria such as *Citrobacter* spp., *Pseudomonas aeruginosa*, *Klebsiella pneumoniae* and *Morganella morganii* have been proven to be susceptible to SSRIs [120,123]. Notably, most of these bacteria are key players in induction of inflammation in the gut [99,100,103,104,105,106,107,108]. This suggests that through their antimicrobial activity, antidepressants might restore a balanced composition of the gut microbiota, and immune response, hence re-establish homeostasis at the gut–brain interphase. Deciphering the actual contribution of the antimicrobial effects of antidepressants for treatment of depression as well as determining the long-term consequences of these effects to gut microbiota composition and their implications to clinical outcomes is crucial for the development of microbiota derived therapeutic alternatives [3,8,120,123].

Taken together, game-changing science is suggesting that depression is not only a result of a deficiency of serotonin and other neurotransmitters in the brain, but could rather start in the gut, via changing the microbiota composition through consumption of processed, nutrient poor diet, which in turn, leads to a state of inflammation, imbalanced levels of neurotransmitters, and eventually depression.

### 3.3. The Dual Effect of Gut Microbiota and Its Metabolites in Depression

Recently, changes in the composition of the gut microbiota have been associated with depressive-like behavior in humans and animal models [124,125]. Decreased levels of bacterial genera *Bifidobacterium* and *Lactobacillus* and increased levels of Streptococcaceae, Clostridiales, Eubacteriaceae and Ruminococcaceae, have been positively correlated with depressive symptoms [124,126]. Kelly et al. have shown that fecal microbiota transplantation from depressed patients to microbiota-depleted rats induced behavioral and physiological changes, leading to anxiety-like behaviors in the recipient animals, as well as alterations in tryptophan metabolism [125]. This suggests that changes in gut microbiota composition could play a causative role in the onset of depression. 

Probiotic therapies have been applied in an attempt to correct for the possible absence of microbiota species capable of exhibiting suitable drivers of a “healthy” behavior. For example, the classical probiotics, Bifidobacteria and Lactobacilli, have been recently suggested as an alternative treatment for anxiety and depressive-like behaviors. Oral administration of a combination of *Lactobacillus helveticus* R0052 and *Bifidobacterium longum* R0175 (Probio’Stick^®^, Lallemand, Montreal, QC, Canada) for a period of one month, has been reported to improve depression, anxiety, and lower the level of the stress hormone cortisol in humans (*n* = 26) [127]. A three-week consumption of a probiotic-containing milk drink that contained *Lactobacillus casei* Shirota, showed improved mood in healthy volunteers (*n* = 124) [128]. Similarly, when healthy male and female participants (*n* = 20) were administered with, either a placebo product or a mixture of several probiotics strains of Bifidobacteria and Lactobacilli over a period of 4 weeks, they exhibited substantially reduced reactivity to sad mood compared to control group [129]. Another small (*n* = 12) placebo-controlled study involving functional magnetic imaging has also demonstrated that a one-month consumption of a fermented food containing *Bifidobacterium animalis* subsp. *lactis*, *Streptococcus thermophilus*, *Lactobacillus bulgaricus*, and *Lactococcus lactis* subsp. *lactis* can influence brain activity as compared to baseline [130]. More recently, *Lactobacillus reuteri* has been described to reduce despair like behavior in mice by inhibiting elevated levels of IDO and reducing peripheral levels of kynurenine [131]. Whether the observed antidepressant effect of probiotics is due to their modulation of an intestinal inflammatory state, restoration of tryptophan metabolism, or reduction in serotonin turnover is still unclear. 

Important to consider is indeed the influence of altered IDO activity and kynurenine pathway metabolism induced by gut microbiota [132]. In the germ-free state, microbial colonization induced the expression of genes encoding IDO, suggesting that gut microbiota activates this enzyme [17,96,132]. Moreover, other bacteria that flourish in an inflammatory environment, in particular *Pseudomonas* genera, can catabolize tryptophan into kynurenine via tryptophan 2,3-dioxygenase, *kynA* and kynurenine formamidase, *kynB* [133] (Figure 1D). Whether intestinal proinflammatory cytokines or any other metabolites have similar effect on induction of *kynA* and *kynB* expression in this bacterium, and subsequent increased levels of downstream metabolites, is still unknown. Additionally, in *Pseudomonas aeruginosa*, kynurenine acts as the main precursor of the *Pseudomonas* quinolone signal, a quorum-sensing signal that regulates numerous virulence genes in these bacteria [133]. This suggests that shifting tryptophan metabolism towards kynurenine during inflammation might result in inducing virulence in *Pseudomonas*, which in turn, causes imbalance in the microbial population, and disruption in the kynurenine and serotonin signaling systems, eventually leading to a state of depression. 

Besides the kynurenine and serotonin arms within tryptophan metabolism, indole represents another important product in this metabolic pathway. Indole and its derivatives are exclusively produced by gut bacterial metabolism of tryptophan, via the tryptophanase (*tnaA*) enzyme [111,134,135]. In their recent rodent study, Jaglin et al. suggested that human subjects, who carry microbiota type dominated by species capable of overproducing indole may be more prone to develop anxiety and mood disorders [136]. The authors mimicked this situation by injecting indole in the cecum of conventional rats. The treated rats showed a dramatic decrease of motor activity, and higher levels of the indole-derivatives oxindole and isatin were detected in the brain. When germ-free rats were colonized with the indole-producing bacterial species *E. coli* to mimic a state of a chronic and moderate overproduction of indole and compared their behavior with that observed in germ-free counterparts mono-colonized with a mutant strain *E. coli*^−Δ*tnaA*^, which is unable to produce indole, only rats colonized with wild-type strain showed anxiety-like behavior suggesting that indole and its metabolites might play a role in developing depression [136]. This study implies a direct mechanism by which the gut microbiota can influence the brain, and result in a state of depression, in this case via the production of the neuro-suppressive indole-derivatives; oxindole and isatin, which are products of gut epithelial or hepatic xenobiotic metabolizing enzymes (Figure 1D). However, another plausible mechanism could be through activation of the vagal afferent fibers in the intestinal mucosa either directly by indole or indirectly via secondary signals whose production could be triggered by indole. Another indole derivative, indole pyruvic acid, was shown to normalize the level of corticosterone in rodent model of depression and this effect was suggested to be due to the production of kynurenic acid in the brain [137]. Altogether, further studies are warranted for a comprehensive understanding of the mechanisms governing the beneficial or detrimental effects of gut microbiota and its metabolites on mood and behavior. 

## 4. Conclusions and Future Perspectives

Given the mounting evidence over the past five years that microbiota play a key role at the gut–brain interphase shows a need to reveal the mechanisms that underpin this interaction in order to close the gap between therapeutic strategies and fundamental science. That the metabolites of the gut microbiota is evident to have a substantial effect on the regulation of immune response, tryptophan metabolism, and serotonin production, a diet characterized by nutrient-poor, energy-dense processed foods can well explain the strong link between depression and this multifaceted crosstalk. Restoring the gut microbiota composition via nutritional interventions could be an indirect strategic tool to treat depression. The use of selective dietary microbial growth substrates could be as beneficial but may result in long-lasting changes of the microbiome compared to the application of probiotic therapies.

Achieving a better understanding of the role of the complex triggers of depression requires further development of analytical approaches, including, metabolomics, to allow unraveling the metabolic dialogue between the microbiota and gut–brain axis. Equally important is the development of reliable models to decipher the complex interactions between the gut microbiota and its products, disruptions in immune response and dietary metabolism, all of which ultimately affect brain functionality, mood and behavior. The use of reductionist animal models has been very helpful in identifying underlying mechanisms in the host–microbe cross talk. However, it is increasingly clear that animal models fall short in translation to humans. Data acquired from large longitudinal human cohorts followed over long period of time, is essential to understand the real-world complexity of these interactions. Currently, there is an exponential growth of large bio-banks holding vast amounts of information about the same individual [138,139,140]. If combined with the state-of-art technologies including bacterial culturomics and individualized organs-on-chips to further understand the underlying causalities and mechanisms, only then we can bridge the gap between basic science and clinical practice and make major advances in personalized medicine.
